# A Transcriptome Analysis: Various Reasons of Adverse Pregnancy Outcomes Caused by Acute *Toxoplasma gondii* Infection

**DOI:** 10.3389/fphys.2020.00115

**Published:** 2020-02-19

**Authors:** Xue Zhou, Xiu-Xiang Zhang, Yasser S. Mahmmod, Jorge A. Hernandez, Gui-Feng Li, Wan-Yi Huang, Ya-Pei Wang, Yu-Xiang Zheng, Xiu-Ming Li, Zi-Guo Yuan

**Affiliations:** ^1^College of Veterinary Medicine, South China Agricultural University, Guangzhou, China; ^2^Key Laboratory of Zoonosis Prevention and Control of Guangdong Province, Guangzhou, China; ^3^Key Laboratory of Zoonosis of Ministry of Agriculture and Rural Affairs, South China Agricultural University, Guangzhou, China; ^4^College of Agriculture, South China Agricultural University, Guangzhou, China; ^5^IRTA, Centre de Recerca en Sanitat Animal, Barcelona, Spain; ^6^Universitat Autónoma de Barcelona, Barcelona, Spain; ^7^Infectious Diseases, Department of Animal Medicine, Faculty of Veterinary Medicine, Zagazig University, Zagazig, Egypt; ^8^College of Veterinary Medicine, University of Florida, Gainesville, FL, United States; ^9^College of Animal Science and Technology, Heilongjiang Bayi Agricultural University, Daqing, China

**Keywords:** *T. gondii*, RNA-Seq, acute infection, abnormal pregnancy, DEGs

## Abstract

**Background:**

*Toxoplasma gondii* (*T. gondii*) is an obligate intracellular parasite, which can affect the pregnancy outcomes in infected females by damaging the uterus, and the intrauterine environment as well as and the hypothalamus resulting in hormonal imbalance. However, the molecular mechanisms underlying the parasite-induced poor pregnancy outcomes and the key genes regulating these mechanisms remain unclear. Therefore, this study aimed to analyze the gene expression in the mouse’s uterus following experimentally-induced acute infection with *T. gondii* RH strain. Three groups of female mice were intraperitoneally injected with tachyzoites as follow; 3 days before pregnancy (FBD6), after pregnancy (FAD6), and after implantation (FID8) as the experimental groups. Another corresponding three groups served as control, were injected with normal saline at the same time. Transcriptome analysis of the total RNA extracted from both infected and non-infected mouse uterus samples was performed using RNA sequencing (RNA-Seq).

**Results:**

The three experimental groups (FBD6, FAD6, and FID8) had a total of 4,561, 2,345, and 2,997 differentially expressed genes (DEGs) compared to the controls. The significantly upregulated and downregulated DEGs were 2,571 and 1,990 genes in FBD6, 1,042 and 1,303 genes in FAD6 and 1,162 and 1,835 genes in FID8 group, respectively. The analysis of GO annotation, and KEGG pathway showed that DEGs were mainly involved in anatomical structure development, transport, cell differentiation, embryo development, hormone biosynthetic process, signal transduction, immune system process, phagosome, pathways in cancer, and cytokine-cytokine receptor interaction pathways.

**Conclusion:**

*T. gondii* infection can induce global transcriptomic changes in the uterus that may cause pregnancy hypertension, destruct the intrauterine environment, and hinder the normal development of placenta and embryo. Our results may help to understand the molecular mechanisms of the acute *T. gondii* infection, which could promote the development of new therapeutics or prophylactics for toxoplasmosis in pregnancy.

## Introduction

Toxoplasmosis is a widespread zoonotic disease caused by the obligate intracellular protozoan parasite, *Toxoplasma gondii* (*T. gondii*). More than 30% of the human population has been exposed to *T. gondii* infections (Hill and Dubey, 2002). The main routes of *T. gondii* transmission are the oral and transplacental transmission However, *T. gondii* can be transmitted via blood transfusions and organ transplantation. *T. gondii* infections are usually asymptomatic but may cause serious complications, such as severe fever in infants, and fatal encephalitis in immunocompromised individuals such as AIDS patients (Luft et al., 1983). Moreover, immunosuppressed *T. gondii*-infected women during pregnancy are susceptible to abortion or stillbirth. Even if the fetus survives, there is a high probability of vertical transmission with subsequently expected serious sequelaein the congenitally infected fetuses such as hydrocephaly, retinochoroiditis, mental retardation, and seizures (Saadatnia and Golkar, 2012).

Females infected with *T. gondii* during pregnancy are three times more likely to experience a problematic pregnancy (Xiao et al., 2010). Stahl et al. (1985) reported a reproductive failure, caused by the hypothalamus dysfunction, in *T. gondii*-infected mice. As well as, these mice showed atrophy of the uterus and ovary, low mature oocytes number and oocyte chromosome aneuploidy, which increase the incidence of infertility (Stahl et al., 1985). At present, the hypothesis about the mechanism of infertility in *T. gondii*-infected women includes endometritis, and expulsion of the fetus. This hypothesis suggests that, during the placental formation, *T. gondii* cysts can be formed in the endometrium inducing non-suppurative inflammation (Remington et al., 1958). While this protozoan is being released, it irritates the placenta and causes miscarriages (Remington et al., 1961). Another hypothesis suggests that persistent infection of *T. gondii* in the hypothalamus results in infertility with follicle damage, and uterine atrophy (Aral et al., 2011). Barbosa et al. (2012) has shown that enoxacin, which is used for endometritis treatment in animals, has also a role in inhibiting the proliferation of *T. gondii*.

Previous research has shown that the incidence of uterine toxoplasmosis in the early stage of pregnancy is less than 6% (Dunn et al., 1999). However, an acute infection in early pregnancy can lead to serious clinical symptoms in 61% of the infected cases (Montoya and Remington, 2008). The offspring of mice infected with *T. gondii* in early pregnancy had a 40% survival rate and more stillbirths (Freeman et al., 2005; Rorman et al., 2006). Mice with acute infection (72 h after intraperitoneal injection) revealed tachyzoites in almost all nucleated cells with abnormal cell morphology in the uterus and placenta (Chen et al., 2003). In experimental acute vaginal infection set-up with *T. gondii* RH strain, tachyzoites and DNA of *T. gondii* were detected through biopsy and PCR assays, respectively in the uterine mucosa, and myometrium as well as the placental chorion of mice (Bayat et al., 2013). Several studies have investigated the effect of acute *T. gondii* infection during pregnancy, however, it focused principally on the immune responses associated with *T. gondii* infection (Nishimura et al., 2017; Xu et al., 2017). To best of our knowledge, research on the evidence of *T. gondii-*associated key genes, which are involved in the adverse pregnancy and disturbance of the pregnancy, is very limited and seldom.

Transcriptome analysis has been a valuable approach for investigating the global biological changes, gene expressions, and the transcription regulations in different body tissues of mice infected with *T. gondii*. For instance, Tanaka et al. used RNA-Seq approach to study the transcriptomic gene expressions in the brain of *T. gondii*-infected mice (Tanaka et al., 2013). Earlier to that, Knight et al. (2005) utilized the transcriptomics to reveal the role of inflammation in *T. gondii*-associated retinochoroiditis. More recently, He et al. (2016b) analyzed the transcriptomic changes in the spleen of infected mice to elucidate the immune responses against *T. gondii* infection. Later, they evaluated the transcriptomic gene expressions in the liver of infected mice during the early stages of *T. gondii* infection (He et al., 2016a). However, data on the global transcriptomic changes in uterine tissues of mouse with acute *T. gondii* infection are highly deficient, despite of its crucial importance.

To elucidate the underlying molecular mechanisms of the adverse pregnancy outcomes associated with the acute *T. gondii* infection, we utilized transcriptome sequencing technology (RNA-Seq) coupled with Bioinformatics approaches to analyze the transcriptomic gene expressions in the uterus of experimentally-infected mice with *T. gondii* RH strain during the acute stage of infection. Full understanding of these mechanisms will promote the development of effective therapeutics and/or vaccines to control and minimize the *T. gondii* infections.

## Materials and Methods

### Animals, Parasite Challenge and Sample Collection

**FIGURE 1:**
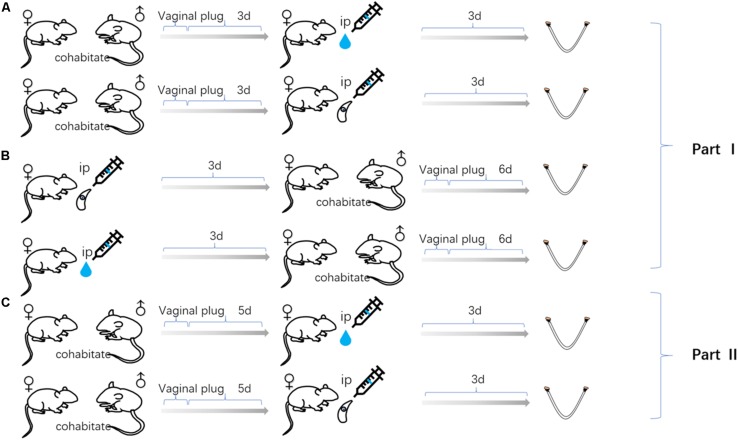
Uterine samples were collected from three mouse models of gestation: **(A)** The female mice infected with *Toxoplasma gondii* tachyzoites 3 days after pregnancy. **(B)** The female mice infected with tachyzoite 3 days before pregnancy. **(C)** The female mice infected with tachyzoite on the fifth day of pregnancy. Mice injected with normal saline (control group) at the same time as every experimental group.

Specific-Pathogen Free (SPF) Kunming mice, purchased from Guangdong Medical Laboratory Animal Center (Guangdong, China), were subjected to carry out this study. Eight-week-old non-pregnant female Kunming mice were randomly divided into six equal groups (20 mice per group). Three groups of female mice were infected with tachyzoite, as follow; 3 days before pregnancy (FBD6; 3 days before cohabiting with male mice at a ratio of 2:1), 3 days after pregnancy (FAD6), and the last group, on the fifth day of pregnancy (FID8; 3 days after implantation). The vaginal plug, the indicator of pregnancy success, was carefully inspected to determine the start of pregnancy. Each mouse in the infected groups was intraperitoneally infected with 80 tachyzoites of *T. gondii* RH strain (Genotype I). Another three corresponding female mice groups, works as control groups, were injected with the normal saline at the same times as the former groups. All experiments were carried out in three replicates. All mice were humanely euthanized by CO2 asphyxiation on the sixth or eighth day of pregnancy for the groups of 3-days before and 3-days after pregnancy infected mice. Whereas, the third group, 3 days after implantation, were euthanized on the eighth day of pregnancy. Uterine tissues were quickly harvested. Each uterine sample was divided into three portions for the subsequent examinations including transcriptome sequencing, qPCR, and follow-up experiments, respectively. Afterward, the uterine samples were rapidly stored at −80°C until subsequent use. Animal experiments were applied in accordance to the standard feeding procedures and experimental procedures of South China Agricultural University. Figure 1 summarizes the time of infection and harvesting of uterine samples in all mice groups.

### mRNA Library Preparation and Sequencing

Experimental methods of the library preparation and RNA-Seq were conducted according to the standard procedures of Illumina. The total RNA was extracted from the preserved uterine samples using RNAiso Plus Total RNA extraction reagent following the manufacturer’s protocol (Takara, Dalian, China). The RNA quality was initially evaluated with 1.0% agarose gel electrophoresis and ultraviolet spectrophotometer (Biotek EPOCH, United States). Following that, the integrity of RNA samples was confirmed by Agilent Bioanalyzer 2100 and RNA 6000 Nano Lab Chip Kit (Agilent Technologies, Santa Clara, CA, United States). RNA integrity number (RIN) over 7.0 was considered as the purity standard. Approximately, 10 μg of total RNA was utilized to isolate Poly (A) mRNA using poly-T oligo-attached magnetic beads (Invitrogen, United States). The product was purified, and the mRNA was segmented into small fragments utilizing divalent cations at elevated temperature. After that, the sliced RNA fragments were reverse-transcribed to establish the final cDNA library according to the recommendations of the mRNA-Seq sample preparation kit (Illumina, San Diego, CA, United States). The average interposer dimension for the paired-end libraries was 300 bp (±50 bp). Finally, the paired-end sequencing was performed by the Illumina Hiseq2000/2500 (LC Sciences, United States) according to the manufacturer’s recommendations.

### Processing of RNA-Seq Data

After the transcriptome sequencing using the Illumina paired-end RNA-Seq methodology, the quality and integrity of the raw reads data were analyzed. The adaptors in the sequenced reads, which have a ratio of base *N* > 5% and nucleotide with Q quality score < 20 were removed. Eventually, clean sequence reads were mapped to the mouse reference genome using TopHat2 aligner and gene transfer format (gtf) annotations data (Mus_musculus.GRCm38.69). A statistical analysis of the three aspects of TopHat2 including the reads statistics, the outline of regional distribution, and the chromosome distribution density was performed. For normalization of transcription profiles, genetic variance analysis was conducted based on the mapping results using the “cuffdiff” command in the cufflinks (Trapnell et al., 2010). The FPKM (Fragments Per Kilobase of exon model per Million mapped reads) value was used to calculate the abundance of gene expression.

### Identification and Bioinformatic Analysis of Differentially Expressed Genes

Differentially expressed genes (DEGs) were identified by the EBSeq package and DESeq package of R software (Anders and Huber, 2010). The *P-values* were calculated using the Benjamini and Hochberg method (Ferreira, 2007). Significant DEGs were identified as those with | log 2 FC (fold-change) | ≥ 1 and an adjusted *P-value* of < 0.05. To understand the biological functions of these genes, firstly; UniProt database was used to expand the biological functions of DEGs. Afterward, we mapped all DEGs to the Gene Ontology (GO) database^1^ and classified these genes into three categories; biological process, molecular function, and cellular component. The Kyoto Encyclopedia of Genes and Genomes (KEGG)^2^ was used to predict molecular function, biological processes and significant DEGs pathways. Finally, the database for Annotation, Visualization, and Integrated Discovery (DAVID) online tool^3^ was used to systematically judge the affected pathways of DEGs.

### Validation of RNA-Seq Results by Quantitative Real-Time PCR (qRT-PCR) Analysis

Gene expression data were verified by qRT-PCR. The total RNA was extracted from the remaining uterine specimen using the RNAiso Plus (Takara, Dalian, China) following the manufacturer’s protocols. The obtained RNA was resuspended in RNase-free water. The purity and concentration of total RNA were determined using ultramicrospectrophotometer (Biotek EPOCH, United States). Ten DEGs were selected to verify the gene expression data by qRT-PCR. Gene-specific qRT-PCR primers were designed, based on reference UniGene sequences, with Premier 5.0 software (Premier Biosoft International, Palo Alto, CA, United States). The genes and primers used in qRT-PCR are shown in Table 1. The first strand cDNA was synthesized by reverse transcription of RNA using RevertAid First Strand cDNA Synthesis Kit (Thermo Fisher Scientific, United States). Quantitative PCR was performed on a Rotor-Gene Q (Qiagen, Hilden, Germany) using AceQ qPCR SYBR^®^ Green Master Mix (Vazyme, Nanjing, China) according to the manufacturer’s instructions. The thermal cycling program included 95°C for 5 min, 40 cycles of 95°C for 10 min, and 30 s of 60°C, followed 95°C for 15 s, 60°C for 1 min, and 95°C for 15 s as a final step. Each sample was examined in triplicate. Relative quantification of specific mRNA levels was performed using the cycle threshold (Ct) 2^–ΔΔCt^ method (Livak and Schmittgen, 2001). For normalization of gene expression, β-actin was used as an internal standard of mRNA expression, and the blank control was used as a reference sample, which was set to a value 1.

**TABLE 1:** Primer design table for qRT-PCR analysis.

	**Gene**	**Description**	**Forward primer (5′–3′)**	**Reverse primer (5′–3′)**
1	Hsd11b2	ENSMUSG00000031891	ATAGCCCTGGTGCCCTAGAA	TCCAGAACACGGCTGATGTC
2	Slc39a4	ENSMUSG00000063354	CTCTGGATCGCCTGGAACTG	CTGTGGCTTGTCAGGTTTGC
3	Ifitm1	ENSMUSG00000025491	ATGGTGGGTGATACGACTGG	GGCACAGACAACGATGACGA
4	Krt8	ENSMUSG00000049382	TATGGGGGACTCACTAGCCC	CAGCTTCCCATCTCGGGTTT
5	Cd24a	ENSMUSG00000047139	CCCACGCAGATTTACTGCAAC	CTGGTGGTAGCGTTACTTGG
6	Cndp2	ENSMUSG00000024644	GTGCCAGACATGATACCGGA	CCCGCCTGGTAATGAGGATG
7	Hsd17b7	ENSMUSG00000026675	GCGTGTAGGAACCTGAGCAA	GCTTGACTTCCTCTGCACCC
8	A2m	ENSMUSG00000030111	AATATCGGTCTTCCGGCCAC	TGTGAGCAGTACGCTTTGGT
9	Stc1	ENSMUSG00000014813	ATAGGAGGCGCACAAATGAG	GGGAGGTGCGTTTGATGTGT
10	Hmgn5	ENSMUSG00000031245	CGACTGTCTGCTATGCCTGT	TGGCTTGGTTTCAGGAATTGG
11	Actb	ENSMUSG00000029580	AAAGAGAAGCTGTGCTATGTTGCT	GCCTCAGGGCATCGGAAC

### Validations of RNA-Seq Results by Western Blotting

Western Blot was used to validate RNA-Seq results at the molecular level, by targeting the protein of two DEGs, Corticosteroid 11-beta-dehydrogenase isozyme 2 (Hsd11b2) and Interferon-induced transmembrane protein 1 (Ifitm1). Firstly, the pre-processed protein samples were run on a 5% stock gel at 80 V with 30 min, separated on a 12% SDS-PAGE in 60 min at 120 V, and then transferred into a nitrocellulose filter membrane (Millipore, Darmstadt, Germany) for 90 min at 150 mA. The membrane was blocked in 0.1% Tween 20-PBS containing 5% skimmed milk powder for 2 h and then incubated with β-actin mouse monoclonal antibodies (1:500 dilution, Neobioscience, Shenzhen, China), specific mouse anti-Hsd11b2 (1:500, Abcam, Cambridge, MA, United States), and anti-Ifitm1 (1:500, Abcam, Cambridge, MA, United States) antibodies overnight. The membranes were washed three times and probed with goat anti-mouse IgG conjugated with horseradish peroxidase (HRP) (Tiangen Biotech, Beijing, China) at 1:2500 dilution. Finally, the membranes were visualized with BeyoECL Plus (Beyotime, Shanghai, China) and the image was taken and analyzed by the Tanon5200 western blotting detection system (Tanon, Shanghai, China).

## Results

### Identification of Expressed Transcripts in the Mouse Uterus Transcriptome

**FIGURE 2:**
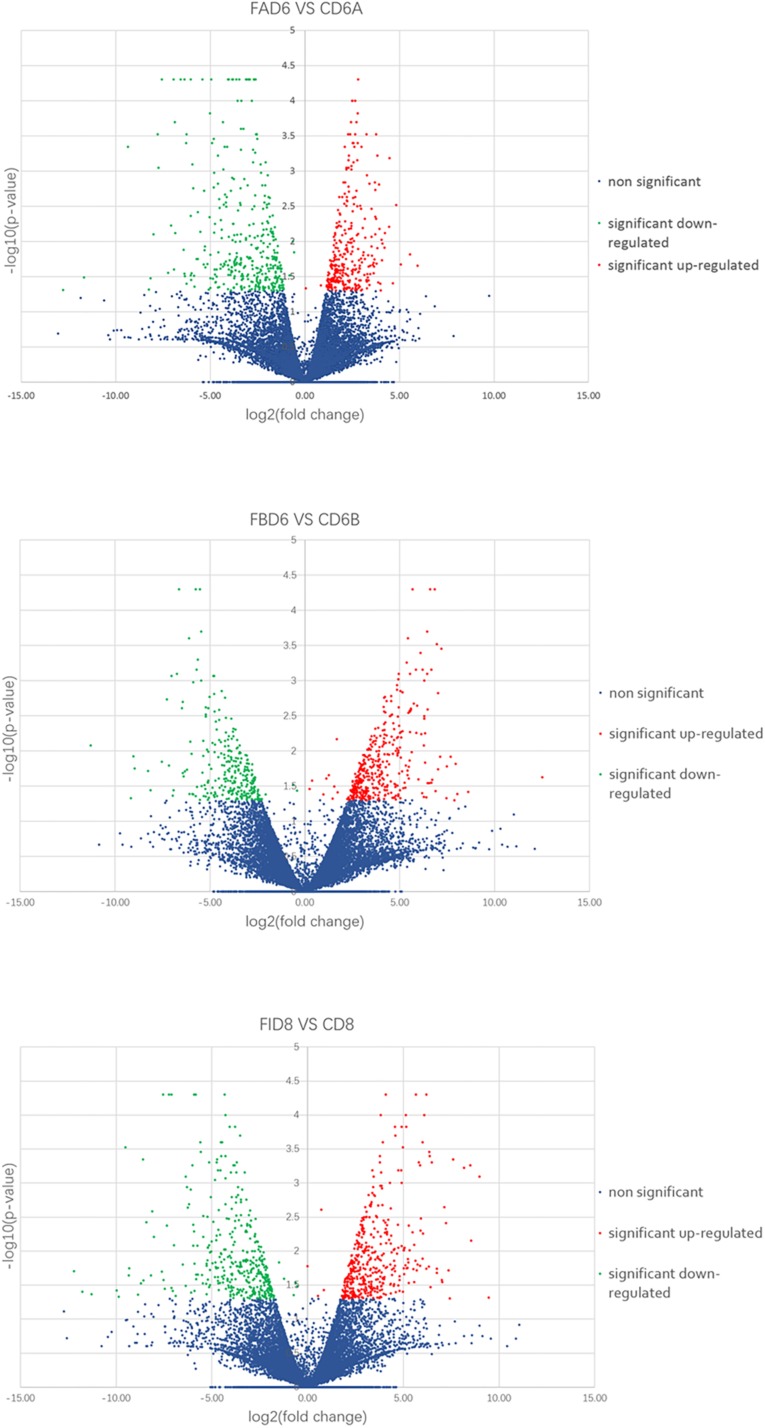
Volcano maps of differentially expressed genes in the uterus of three infected mice groups compared to control groups. Significantly upregulated and downregulated genes are shown as a red and green dot, respectively. The blue dot represents genes without a significant difference in the expression level.

**FIGURE 3:**
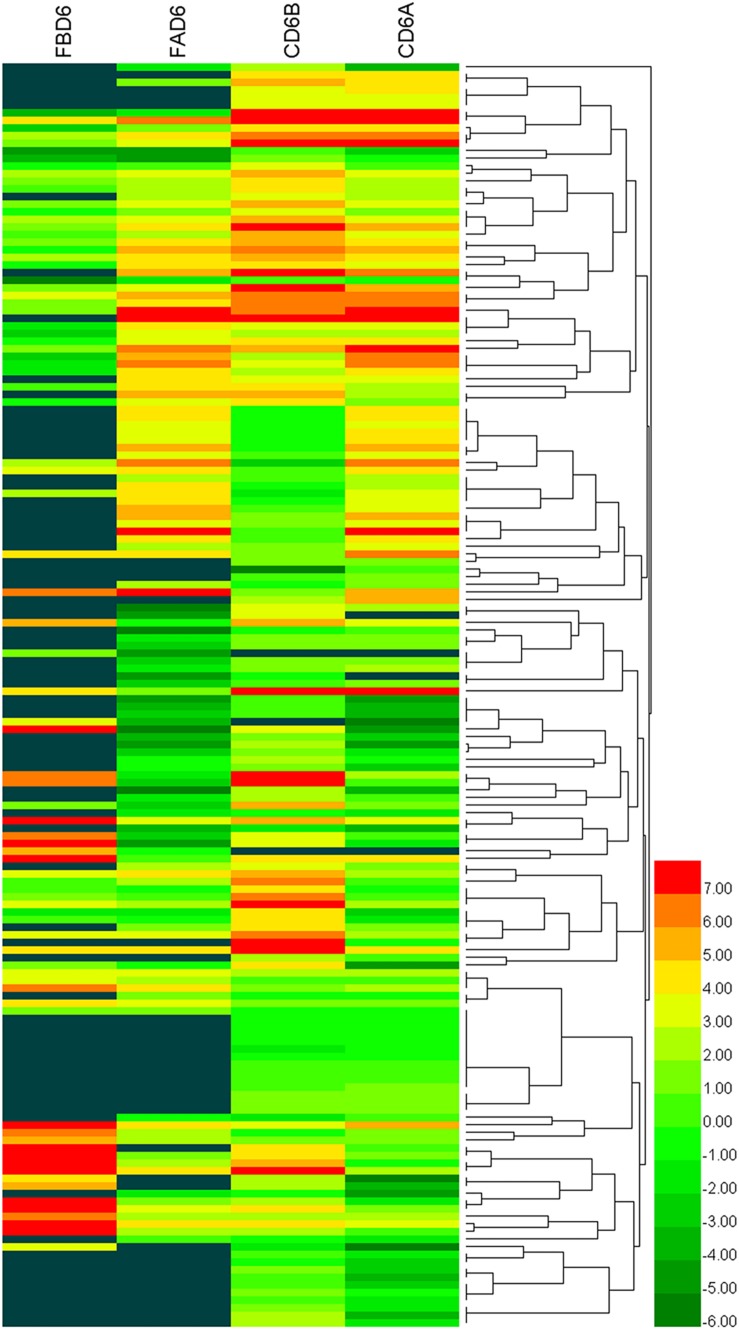
Heat maps representing hierarchical clustering analysis of differentially expressed genes in the uterus of mice infected with *T. gondii* tachyzoites 3 days before pregnancy (FBD6) and 3 days after pregnancy (FAD6). Each row refers to the expression level of each gene in the various groups, and each column refers to the expression level of all DEGs in each group.

We established six different groups of mice, three experimental and three control groups, including mice infected with *T. gondii* 3 days after groups day of pregnancy (FAD6), mice infected with *T. gondii* 3 days before pregnancy (FBD6), mice infected with *T. gondii* on the fifth day of pregnancy (FID8), mice injected with normal saline (NS) 3 days after pregnancy (CD6A), mice injected with NS 3 days before pregnancy (CD6B), and lastly, mice injected with NS on the fifth day of pregnancy (CD8). Each group was tested in triplicates. The total RNA harvested from the uterus of all mice groups was subjected to high-throughput sequencing on Illumina Hiseq2000/2500. The number of the obtained raw reads was ranged from 56,804,874 (CD6B1 group) to 66,241,238 (FAD63 group). After filtration, the average number of clean reads of each mouse group was over 48,000,000, where 82.32–98.90% could be aligned to the reference mouse genome (Table 2). The sequence data of each group had more than 6G and Q20 > 99%. Therefore, the requirements of subsequent analysis were fulfilled. A filter criteria set at | log2 fold change| > 1 and *P-value* ≤ 0.05 to determine whether the difference was significant or not (shown in Supplementary Data Sheets 1, 2). Our findings showed that the FBD6 group produced a total of 4,561 DEGs compared to the control group B (CD6B). Out of them, 2,571 and 1,990 genes were identified as upregulated and downregulated, respectively. A total of 2,345 genes showed significant expression differences between the FAD6 group and its corresponding NS-treated group (CD6A). Out of this number, 1,042 and 1,303 genes were identified as upregulated and downregulated genes, respectively. As f for the third and last groupFID8 and its corresponding control group (CD8); 2,997 genes showed significantly different expression, where 1,162 and 1,835 of them were identified as upregulated and downregulated, respectively. The details of the top 10 DEGs in *T. gondii*-infected mouse uterine samples are shown in Table 3. Volcanic maps showed the distribution of gene expression differences between the experimental and control groups. The maps highlight the genes with an absolute fold change of ≥1 and an adjusted *p*-value of <0.05 after infection (Figure 2). Figure 3 shows a hierarchical clustering analysis of genes expressed in the mouse uterus infected with *T. gondii* in the various groups.

**TABLE 2:** Summary of clean reads mapped to the reference genome of mouse.

**Sample**	**Raw date read**	**Base**	**Valid date read**	**Base**	**Valid% read**	**Q20%**	**Q30%**	**GC%**
CD6A-1	58748442	8.81G	48364460	7.25G	82.32	99.20	96.44	49.00
CD6A-2	59452796	8.92G	48959376	7.34G	82.35	99.52	97.21	51.00
CD6A-3	58563520	8.79G	48696972	7.30G	83.15	99.13	95.98	49.00
FBD6-1	59970362	9.00G	57652548	8.64G	96.14	99.39	97.09	50.00
FBD6-2	57907066	8.69G	56827140	8.52G	98.14	99.57	95.75	50.00
FBD6-3	56905122	8.54G	54152732	8.12G	95.16	99.22	96.17	49.00
FAD6-1	65608270	9.84G	57597380	8.64G	87.79	99.55	96.49	50.00
FAD6-2	64061528	9.65G	55865012	8.38G	87.21	99.15	95.86	51.00
FAD6-3	66241238	9.94G	58153066	8.72G	87.79	99.63	97.94	50.00
CD6B-1	56804874	8.52G	49590640	7.44G	87.30	99.66	97.93	49.00
CD6B-2	57290458	8.59G	50295274	7.54G	87.79	99.38	96.92	49.00
CD6B-3	57516602	9.63G	50243946	7.54G	87.36	99.62	97.31	50.00
CD8-1	51722480	7.76G	51153928	7.67G	98.90	99.68	96.19	50.00
CD8-2	54269660	8.14G	53547754	8.03G	98.67	99.69	97.05	49.50
CD8-3	48768472	7.32G	48078448	7.21G	98.59	99.69	96.90	49.00
FID8-1	44641116	6.70G	43937778	6.59G	98.42	99.69	96.97	50.00
FID8-2	53513324	8.03G	52647430	7.90G	98.38	99.65	96.94	50.00
FID8-3	49853240	7.48G	48944858	7.34G	98.18	99.75	97.42	49.50

**TABLE 3:** Partial list of differentially expressed genes in *T. gondii*-infected mice uterus.

	**Gene symbol**	**Description**	**Log2 (fold-change)**			**Differential Expression**
			**FAD6/CD6A**	**FBD6/CD6B**	**FID8/CD8**	
1	Hsd11b2	Hydroxysteroid 11-Beta Dehydrogenase 2	2.40061	3.47496	2.71847	Up
2	Slc39a4	Solute Carrier Family 39 (Zinc Transporter), Member 4	2.72085	4.63169	1.28504	Up
3	Ifitm1	Interferon-induced transmembrane protein 1	1.75738	3.59516	-0.23735	Up (Down in FID8/CD8)
4	Krt8	Keratin, type II cytoskeletal 8	1.01272	3.09309	3.46933	Up
5	Cd24a	Signal transducer CD24	1.29161	1.19154	3.07802	Up
6	Cndp2	Cytosolic non-specific dipeptidase	1.33972	3.14075	2.66401	Up
7	Hsd17b7	3-keto-steroid reductase	−5.40267	−6.46531	−4.46804	Down
8	A2m	Alpha-2-macroglobulin-P	−3.80101	−5.8787	−5.92206	Down
9	Stc1	Stanniocalcin-1	−2.66361	−3.95738	−2.45878	Down
10	Hmgn5	High mobility group nucleosome-binding domain-containing protein 5	−1.26749	−3.44105	−2.30626	Down

### GO Enrichment of Differentially Expressed Genes

**FIGURE 4:**
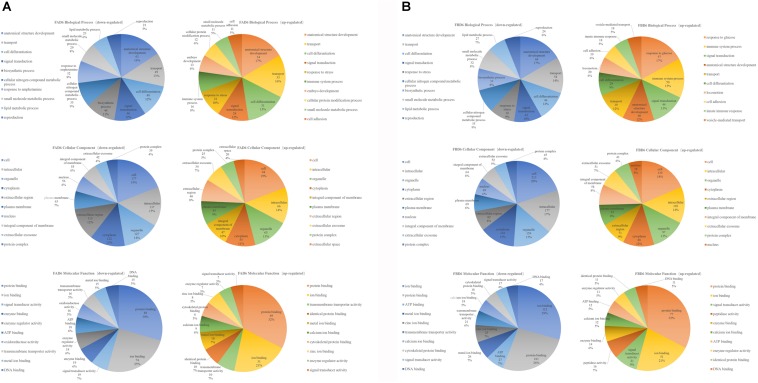
Gene Ontology (GO) distribution of differentially expressed genes of the uterus transcriptome. The top 10 enriched GO terms under biological process, cellular component and molecular function in **(A)** mice infected with *Toxoplasma gondii* 3 days after pregnancy (FAD6) and **(B)** mice infected with *T. gondii* 3 days before pregnancy (FBD6).

The top 10 enriched GO terms under each category in FAD6 and FBD6 groups are shown in Figure 4. In comparison to the control group, the upregulated genes in the FAD6 were annotated in 1,047 GO terms, which were mainly related to biological processes (such as anatomical structure development, transport, cell differentiation, response to stress), cellular components (such as cellular, intracellular, organelle and cytoplasmic component), and molecular functions (such as protein binding, ion binding, transmembrane transporter activity, and cytoskeletal protein binding). The number of GO terms of downregulated genes were 1,622. The most common identified biological process were lipid, small molecule, and cellular nitrogen compound metabolic processes, anatomical structure development, transport and response to amphetamine. The downregulated GO terms categorized under cellular component were similar to the functional classification of the upregulated genes. As for the molecular function, most of the downregulated genes were related to protein and enzyme activity besides binding to protein and ions. In general, there were 21 significant DEGs involved in embryonic development and one gene associated with embryo implantation in the FAD6 group (Figure 4A).

Comparing FBD6 group to the control group, the upregulated genes involved 1,311 GO terms, whereas the downregulated genes were related to 1,744 GO terms. As for the biological process GO terms, the upregulated genes were mainly involved in glucose, immune system process, and signal transduction. Meanwhile, the downregulated genes were mostly related to anatomical structure development, transport, and cell differentiation. In the term of cellular component, the traits of FBD6 vs. CD6B groups were almost similar to those of FAD6 vs. CD6A groups. In respect to the molecular function, the DEGs showed mainly functions related to protein, ion and ATP binding (Figure 4B).

**FIGURE 5:**
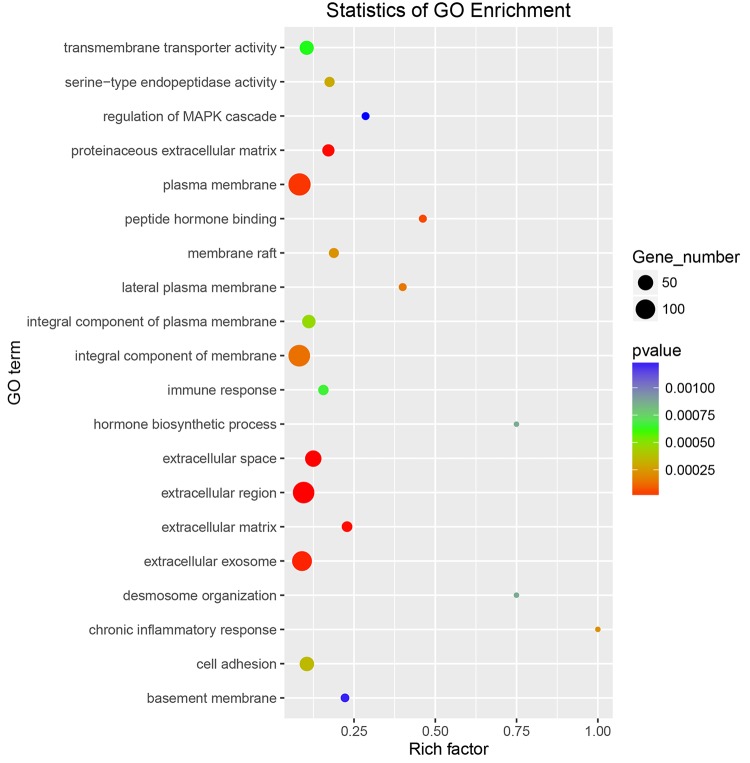
Gene Ontology enrichment scatter plot for differentially expressed genes in the uterus of mice infected with *T. gondii* on the fifth day of pregnancy (FID8).

To explore whether the key genes related to embryo implantation and development are affected by inducing the experimental infection in the pregnant mice with *T. gondii* after implantation or not, we performed GO function classification of the DEGs in FID8 group using the scatter plot (Figure 5). As for the cellular component, DEGs were mostly associated with the plasma membrane, the integral component of the membrane, and extracellular region. Most DEGs were involved in peptide hormone binding, transmembrane transporter activity, and serine-type endopeptidase activity molecular functions. Moreover, DEGs were related to the central biological processes such as hormone biosynthetic process and immune response.

### KEGG Enrichment Analysis

**FIGURE 6:**
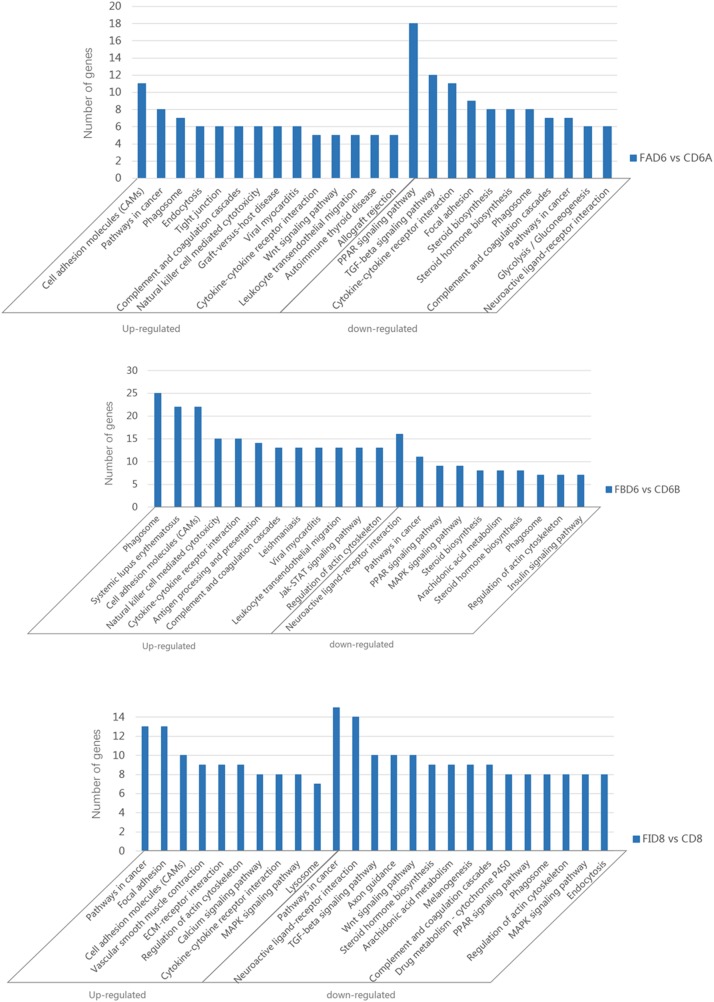
Significantly enriched pathways in the uterus of *T. gondii*-infected mice. [**A** (Upper), **B** (Medium), and **C** (Lower)] represent KEGG pathway analysis of DEGs for FAD6 vs. CD6A, FBD6 vs. CD6B and FID8 vs. CD8 group, respectively.

Enrichment analysis of the DEGs in the uteri of the infected versus the control groups’ pairs was performed by using the KEGG database, where the pathways that play an important role in the infection process can be furtherly explored. Of the 166 identified enriched pathways in the group FAD6 vs. CD6A, upregulated DEGs enriched in 107 pathways, and 136 pathways were enriched by downregulated DEGs. The top five enriched upregulated pathways were cell adhesion molecules (CAMs), pathways in cancer, phagosome, endocytosis, and tight junction. The top five identified enriched downregulated pathways were PPAR signaling pathway, TGF-beta signaling pathway, cytokine-cytokine receptor interaction, focal adhesion, and steroid biosynthesis (Figure 6A).

Of the 175 identified enriched pathways in the FBD6 vs. CD6B group, 128 and 149 pathways were enriched with upregulated and downregulated DEGs, respectively. The major five enriched upregulated pathways were phagosome, systemic lupus erythematosus, cell adhesion molecules, cytokine-cytokine receptor interaction. The major five enriched downregulated pathways involved neuroactive ligand-receptor interaction, pathways in cancer, PPAR signaling pathway, MAPK signaling pathway, and steroid biosynthesis (Figure 6B).

In the FID8 vs. CD8 group, 169 pathways were enriched, including 134 upregulated DEGs enrichment pathways and 145 downregulated DEGs enrichment pathways. The top five enriched upregulated pathways were involving pathways related to cancer, focal adhesion, cell adhesion molecules (CAMs), vascular smooth muscle contraction, and ECM-receptor interactions. However, the top five enriched downregulated pathways were pathways related to cancer, neuroactive ligand-receptor interaction, TGF-beta signaling pathway, axon guidance, and Wnt signaling pathway (Figure 6C).

### Validation of Ten Differentially Expressed Genes by qRT-PCR

**FIGURE 7:**
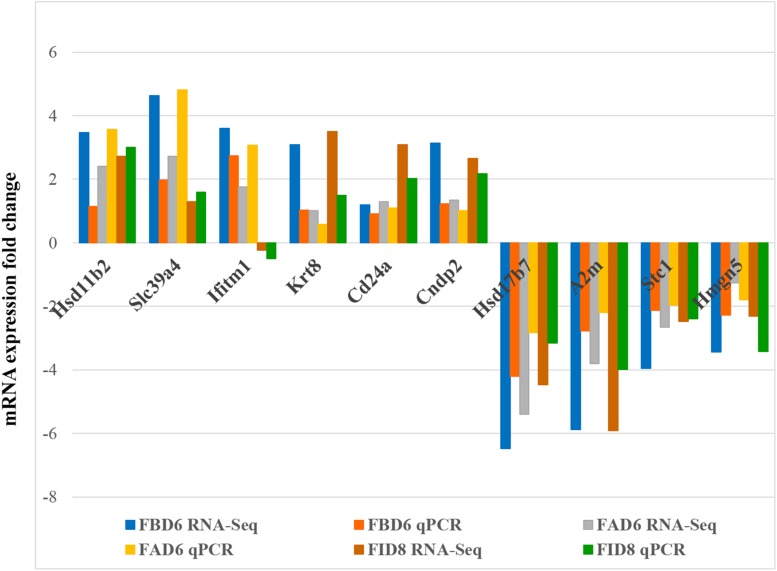
Validation of the relative abundance of 10 DEGs by q-PCR. The *x*-axis represents different DEGs in the uterus from different infected mouse groups and the *y*-axis represents the fold change in mRNA expression.

In order to verify the reliability of RNA-Seq results, we selected 10 DEGs with different expression trends for qRT-PCR. Six genes, Hsd11b2, Slc39a4, Ifitm1, Krt8, Cd24a, and Cndp2 were upregulated, whereas Hsd17b7, A2m, Stc1, and Hmgn5 were downregulated. These results were consistent with the results of RNA-Seq (Figure 7).

### Verification of Hsd11b2 and Ifitm1 Expression Levels by Western Blotting

**FIGURE 8:**
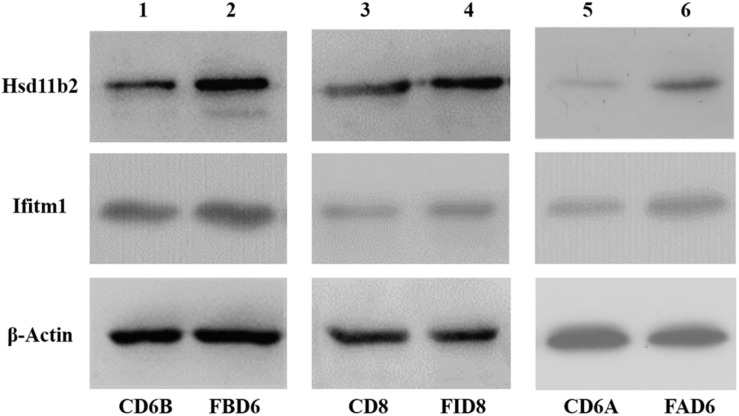
Validation of expression level of Hsd11b2 and Ifitm1 in all three experimental groups by Western blot analysis.

After analyzing the preliminary data and searching relevant literature, Hsd11b2 (UniprotKB: P51661) and Ifitm1 (UniprotKB: Q9D103) were selected as the research targets to explore their role in *T. gondii* infection. Therefore, we verified the expression changes of its protein level by western blot (Figure 8). The results were consistent with the sequencing results of the transcriptome, where these two proteins were overexpressed.

## Discussion

In this study, all the three experimental groups showed pregnancy failure confirming the adverse effect of *T. gondii* infection on pregnancy. Thus, we investigated the causes of *T. gondii*-induced pregnancy failure by analyzing the transcriptomic changes in the uterus of mice with acute *T. gondii* infection. A total of 4,561, 2,345, and 2,997 DEGs were identified in FBD6, FAD6, and FID8 groups compared to the corresponding control groups. Validation of our RNA-Seq results by qRT-PCR and Western blotting confirmed the differential expression, indicating that the RNA-seq results were reliable and subsequent further experiments and analyses can be conducted.

The mechanism of poor pregnancy outcome, associated with *T. gondii* infection, can be discussed from two aspects, the DEGs, and the enrichment pathways. In respect to DEGs, Hsd11b2 gene was overexpressed in infected groups compared to controls. The Hsd11b2 gene is a marker for pregnancy hypertension (Bicho et al., 2012; Majchrzak-Celinska et al., 2017). When the Hsd11b2 gene is overexpressed, it can cause placental dysfunction, especially in early-onset preeclampsia. On top of that, it might affect the embryonic development, and cause even placental abruption (Majchrzak-Celinska et al., 2017). Therefore, pregnant women who are infected with *T. gondii* might have pregnancy hypertension and experience pregnancy failure. A gene named Ifitm1 was also upregulated in the uterus of infected mice. This gene is associated with endometrial tumor when it is overexpressed, thus it is a marker for endometrial tumor. Rojas et al. (2012) demonstrated that endometrial tumor can cause pregnancy failure. Kuang et al. (2009) concluded that after embryo implantation, the chemokine (C-X-C motif) ligand 14 (CXCL14) gene can promote the growth and development of trophoblast. However, CXCL14 gene was significantly downregulated in our transcriptome analysis, which may explain the adverse pregnancy outcome associated with *T. gondii* infection, but perhaps require further experimental confirmation on protein level.

**FIGURE 9:**
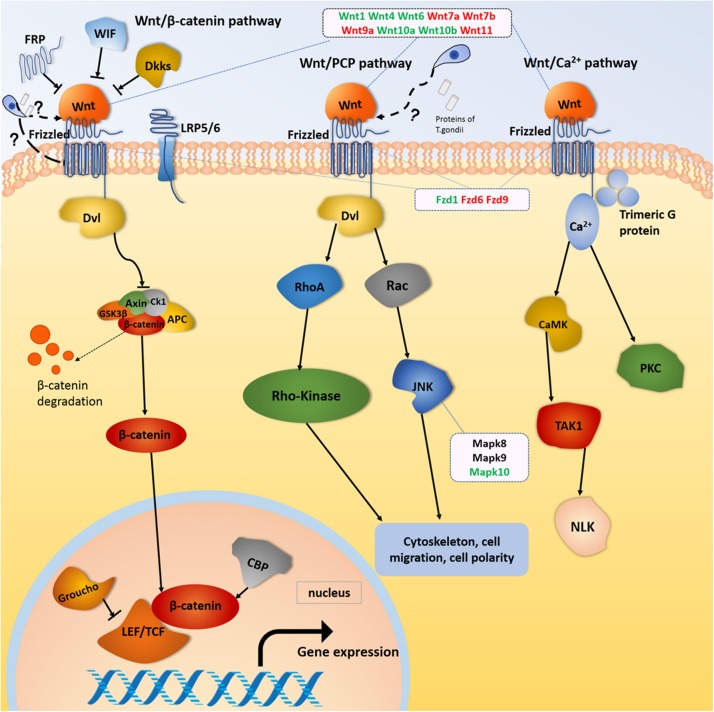
Wnt signaling pathway. Red and green color for genes indicate the upregulation and downregulation, respectively.

The mostly enriched DEGs and pathways in the uterus of the three experimental groups were identified and the signal pathways of pre-implantation and post-implantation were separately analyzed. Our results revealed that the number of DEGs in the cancer pathways was ranked in the top position of all the enrichment pathways. The cancer pathways included five DEGs (Wnt, Frizzled, CBL, JNK, and PPFP), which were shared by the three experimental groups and involved in the three related pathways, which are the wingless-type MMTV integration site family (Wnt) signaling pathway, the phosphatidylinositol 3-kinase-protein Kinase B (PI3K-Akt) signaling pathway, and the peroxisome proliferator-activated receptor (PPAR) signaling pathway. Among them, the DEGs related to the Wnt signaling pathway were the most abundant. The proteins expressed by the Wnt family genes are secreting morphogens, which play an important role in the growth and development of embryos. Such proteins and its products participate in the tissues and organs morphogenesis and the embryonic development and cell division (Moon et al., 2004). The Wnt signaling pathway includes three major transduction pathways (Figure 9), which regulate the growth and development of embryos. These three major transduction pathways are the Wnt/β-catenin classical pathway, the c-Jun NH2-terminal kinases (JNK) gene-mediated and participating planar cell polarity (PCP) pathway, and the Ca-mediated Wnt/Ca^2+^ pathways (Xing et al., 2003). In mice, the Wnt/β-catenin signaling pathway is essential for hormone-mediated uterine growth and implantation (Hou et al., 2004; Mohamed et al., 2005). This is agreement with previous research (Chen et al., 2009), which confirmed the important role of Wnt/β-catenin pathway in embryonic development, pre/post-implantation, and decidualization of the uterus.

Our results showed that Wnt gene expression was altered during embryonic development in female mice infected with *T. gondii.* Wnt gene is not only related to the estrous cycle and steroid hormone levels, but also closely related to the morphological changes regulated by intrauterine epithelium (Park et al., 2011). The Wnt1 gene plays an important role in the development of the embryonic brain and central nervous system (Liu et al., 2005). In our study, the expression of Wnt1 genes in FBD6 and FID8 were inhibited. We believe that inhibition of the Wnt1 gene expression in early pregnancy by *T. gondii* infection had an adverse effect on embryonic development resulting in pregnancy failure. Wnt4 is important for the development of the female reproductive system, and it can be expressed in human trophoblast cells and the uterus of pregnant mice to regulate blastocyst implantation (Tranguch et al., 2005). Knocking out Wnt4 leads to embryo implantation and decidual abnormalities (Franco et al., 2011). Wnt4 expression in endometrial cancer is lower than that in normal tissues (Bui et al., 1997). In our study, Wnt4 was downregulated in the experimental groups with acute *T. gondii* infection. We hypothesize that acute *T. gondii* infection can affect the decidualization reaction and destroy the endometrium environment. Thus, it will hinder the implantation of embryos through reducing the expression of Wnt4. Wnt7a plays many roles, for instance, it stimulates embryo-implantation-associated responses, upregulates β-catenin in the nucleus of uterine epithelial cells, and activates the Wnt/β-catenin signaling pathway as well as improves embryo implantation success rates (Zhang et al., 2009). Wnt7a is also a tumor suppressor gene that inhibits cell proliferation and promotes apoptosis (Li et al., 2013; Bikkavilli et al., 2015). The mutation of Wnt7b can cause placental dysplasia and fetal death in mice (Biechele et al., 2011). In this study, the Wnt7a and Wnt7b gene were upregulated in all experimental groups compared to uninfected pregnant mice. Therefore, we hypothesize that the proliferation effect induced by *T. gondii* looks like the effect of cancer cells. In other words, acute *T. gondii* infection increases the expression of Wnt7a, which in turn, hinders the embryonic cell multiplication, and stimulates the apoptosis process. We think this fact beside other factors could explain the underlying mechanism of adverse pregnancy outcomes associated with acute *T. gondii* infection.

Frizzleds (seven-transmembrane proteins) act as receptors for the Wnt proteins and are involved in many functions such as regulation of embryonic development, cell differentiation, proliferation, apoptosis, and inflammation. The Frizzled gene family “Fzd9, Fzd6, and Fzd1genes,” were differentially expressed in the uterus of our experimental groups (FBD6, FAD6, and FID8). The former studies have shown that Fzd1 and Wnt7a combine to activate the Wnt classical pathway and play a key role in embryonic lung development. Fzd6 is expressed in endometrial epithelium and stromal cells, but it cannot be regulated directly by ovarian steroid hormones. Thus, the expression of Fzd6 is constant during the secretory and proliferative phases (Tulac et al., 2003). Therefore, we suggest that the differential expression of Frizzled genes in our study is caused by the acute infection of *T. gondii*. This finding was also consistent with the claim that the Wnt/Frizzled signaling pathway is involved in the inflammatory process (Neumann et al., 2010). We hypothesize that the Wnt genes expression is strongly influenced by *T. gondii* acute infection, which result in activation of the classical and non-canonical pathways of Wnt signaling pathway, and eventually altering the early embryonic development, the endometrial formation, and decidualization.

On the other hand, the analysis of the signaling pathways in mice infected with *T. gondii* after embryo implantation (FID8 group) showed that the infection had a marked influence on the vascular endothelial growth factor (VEGF) signaling and Wnt signaling pathways. In normal pregnancy, David (2017) demonstrated that placenta angiogenesis, and vascular expansion are enhanced by placental growth factor (PIGF), VEGF and insulin growth factor (IGF). However, VEGF was downregulated in our *T. gondii*-infected groups, indicating that it is responsible for the blockage of angiogenesis in placenta and even, miscarriage. As for the Wnt signaling pathway, we noticed that infection with *T. gondii* decreased the expression of glycogen synthase kinase beta (GSK-β), and β-catenin, which in turn, affect the neural development of the embryo. Wang et al. (2017) treated a group of mice with celecoxib and they found that the expression of GSK-β was changed, and β-catenin was decreased, which resulted in dysplasia of the fetus’ nerve cells. We propose that the reasons for pregnancy failure can be argued by many factors, which take place after the embryo implantation. In this regard, we think *T. gondii* infection can damage the placental tissues and impair the normal development of the embryo.

Concerning the time before the embryo implantation, we found that DEGs were mostly related to cytokines pathway. Transforming growth factor beta (TGF-β) is highly expressed in the normal endometrium epithelium and trophoblastic layer. It plays an important role several process such as endometrial inflammation, maternal support for embryonic development, immune-regulating maternal-fetal interface, and maintaining normal pregnancy. In this study, TGF gene family and the cytokines pathway were downregulated in the infected groups compared to the control. This is consistent with previous studies, which reported that downregulation of TGF could cause abortion or fetal death (Pazmany and Tomasi, 2006; Norris et al., 2011). Therefore, we conclude that infection with *T. gondii* affects the development and implantation of the embryo.

KEGG pathway analysis of DEGs showed upregulation of pathways related to diseases in infected mice such as systemic lupus erythematosus, leishmaniasis, viral myocarditis, and autoimmune thyroid disease. This finding indicates that the abortion caused by *T. gondii* infection during pregnancy is complicated, and may not be exclusively caused by *Toxoplasma* infection, where other diseases may be involved as a secondary sequelae of *T. gondii* infections. Previous research showed a close relationship between *T. gondii* infection and the occurrence of systemic lupus erythematosus in 1990 (Wilcox et al., 1990) and rheumatoid arthritis (Fischer et al., 2013).

## Conclusion

The adverse pregnancy caused by acute toxoplasmosis can be attributed to three factors: (i) direct injury to tissues and cells due to *T. gondii* proliferation, (ii) disruption of the early maternal uterine environment, which impair the normal embryo implantation, and (iii) poor development of embryos before and after the implantation. Our finding indicates that acute *T. gondii* infection altered the Wnt genes expression and signaling pathway, leading to the failure of early embryonic development, endometrial formation, and decidualization. Acute *T. gondii* infection is disturbing the normal implantation of the embryo. Acute infection of *T. gondii* is associated with gestational hypertension, systemic lupus erythematosus, and possibly endometrial tumor resulting in adverse effects on the pregnancy outcome. Our findings uncover the role of the acute *T. gondii* infection and associated mechanisms in the pregnancy failure which could promote the development of a new treatment for toxoplasmosis in pregnancy.

## Data Availability Statement

The sequencing data generated in this study has been deposited into BioProject (accession no. PRJNA593831, https://www.ncbi.nlm.nih.gov/bioproject/PRJNA593831).

## Ethics Statement

Experimental animals were handled according to the Animal Ethics Procedures and Guidelines of the People’s Republic of China. All animal experimental procedures were reviewed and approved by the Experimental Animal Welfare Ethics Committee, the Laboratory Animal Center of Guangdong Province (Reference No. 2016-10).

## Author Contributions

X-ML and Z-GY conceived and designed the experiments. XZ performed the experiments, analyzed the data, and wrote the manuscript. X-XZ, JH, and YM revised the manuscript. XZ, G-FL, W-YH, Y-PW, and Y-XZ contributed to data collection and laboratory work. All authors read and approved the final manuscript.

## Conflict of Interest

The authors declare that the research was conducted in the absence of any commercial or financial relationships that could be construed as a potential conflict of interest.

## Footnotes

1 http://www.geneontology.org/

2 http://www.kegg.jp/kegg/

3 http://david.abcc.ncifcrf.gov/

## Abbreviations

CD6A: mice injected with normal saline (NS) 3 days after pregnancy
CD6B: mice injected with NS 3 days before pregnancy
CD8: mice injected with NS in the fifth day of pregnancy
DEGs: differentially expressed genes
FAD6: mice infected with *T. gondii* 3 days after pregnancy
FBD6: mice infected with *T. gondii* 3 days before pregnancy
FID8: mice infected with *T. gondii* on the fifth day of pregnancy.
